# Enhancing Emergency Nurses' Disaster Nursing Ability and Psychological Resilience: A Randomized Controlled Trial

**DOI:** 10.1155/2023/6108057

**Published:** 2023-11-27

**Authors:** Lin Lan, Meichi Zhou, Ling Wang, Xiaoli Chen, Min Dai, Jianna Zhang

**Affiliations:** ^1^Department of Emergency Medicine, West China Hospital, Sichuan University/West China School of Nursing, Sichuan University, Chengdu, China; ^2^Institute of Disaster Medicine, Sichuan University, Chengdu, China; ^3^Nursing Key Laboratory of Sichuan Province, Chengdu, China; ^4^Nephrology and Urology Ward West China Hospital, Sichuan University/West China School of Nursing, Sichuan University, Chengdu, China

## Abstract

**Objective:**

This study aimed to investigate emergency nurses' disaster nursing ability and psychological resilience, validate the effectiveness of a training system for disaster nursing ability based on psychological resilience, and verify the relationship between psychological resilience and disaster nursing ability among emergency nurses.

**Methods:**

A training system was developed to enhance psychological resilience and disaster nursing ability. A multicenter, randomized controlled experiment was conducted in five Grade III hospitals in Sichuan Province. A total of 93 emergency nurses were randomly assigned to the following three groups: the blank group, intervention group, and control group. The corresponding interventions were as follows: no intervention, psychological resilience, and nurses' disaster nursing ability training. Personal information forms, the Connor–Davidson Resiliency Scale, and the Nurses' Disaster Nursing Ability Assessment Scale were used in the survey.

**Results:**

There was no significant difference in the scores of psychological resilience and disaster nursing ability in the blank group in the pretest and posttest (*P* > 0.05). The disaster nursing ability of both the intervention and control groups significantly improved in the posttest (*P* < 0.05). Compared with the control group, the intervention group demonstrated significant improvement in psychological resilience in the posttest (*P* < 0.05). However, there was no statistical difference in psychological resilience scores in the control group in the pretest and posttest (*P* > 0.05).

**Conclusion:**

The study concluded that the psychological resilience and disaster nursing ability of emergency nurses could be enhanced through the implemented training system.

## 1. Introduction

In recent years, natural disasters have been occurring frequently and nurses have emerged as the largest rescue force in disaster emergency teams. Nurses play a crucial role in disaster prevention, mitigation, and postdisaster recovery. However, some disaster-response nurses have experienced psychological issues such as acute stress disorder and post-traumatic stress disorder during or after rescue operations [1]. Better psychological resilience may prevent the incidence of post-traumatic stress disorder [2].

Psychological resilience refers to an individual's capacity to adapt and recover from adversity, trauma, or significant stress and the ability to maintain stable functioning, meet challenges, and prevail in the face of adversity [3]. Psychological resilience was first proposed by a group of child psychologists in the 1970s [4] and later further explained by scholars, gradually attracting more attention from people [5]. A high level of resilience was cited as being crucial for nurses to succeed professionally and manage workplace stressors [6]. In a review of studies, health workers responded to crises in the face of disaster by communicating with family and friends and through prayer, meditation, and distraction to enhancing psychological resilience [7]. Nurturing nurses' growth, resilience training, fostering mindfulness practice, cognitive reframing, and keeping work-life balance were identified to enhance and build nurses' resilience [8–10]. Enhancing nurses' psychological resilience can effectively improve their ability to respond to adverse events, ensuring high-quality disaster medical rescue and promoting nurses' mental well-being [11]. Currently, most studies focus on assessing the levels of psychological resilience among nurses and investigating the influential factors [12]. However, there remains a dearth of intervention measures to enhance nurses' psychological resilience. First-aid skills of emergency nurses are an important factor of resilience. In the prior research, training programs that encompass psychological knowledge, professional expertise, and skills are considered the main strategies to improve psychological resilience among nurses [8]. In a review by Professor Mao, it was mentioned that nurses with strong resilience often have sufficient disaster rescue capabilities [13]. The level of psychological resilience is also an important characteristic of core competencies in disaster nursing [14]. However, at present, there have been no reports on training that combines psychological resilience with disaster nursing ability. In this study, we assumed that training in disaster emergency skills can also enhance the resilience of nurses.

Research has indicated that the core competencies of emergency nurses in disaster care are at a moderate level [15, 16] and the nurses' disaster preparedness is unsatisfactory; disaster rescue ability can affect nurses' resilience. The aim of this study was to investigate the resilience and disaster rescue ability of emergency nurses and test whether resilience interventions and disaster skills training could improve resilience of emergency nurses. Chinese scholars have integrated the core competencies of International Council of Nurses (ICNs) in disaster care. Chinese researchers extracted four modules, namely, prevention and preparedness capabilities, emergency rescue capabilities, resource allocation and management capabilities, and psychological support and health education capabilities. To improve the disaster nursing ability and psychological resilience level of emergency nurses, the research team planned to create a training course for nurses' disaster preparedness, which contained rescue ability associated with psychological resilience.

### 1.1. Methodology

This was a multicenter, randomized controlled trial that used the e-questionnaire survey method to collect data. The project team issued recruitment notices to recruit hospitals to participate. The inclusion criteria for the research centers were as follows: (1) emergency departments of Sichuan Provincial Third Grade A hospitals, (2) the number of emergency department nurses was more than 30, and (3) the head of the research center voluntarily joined this study after learning about the research plan of the project.

Random allocation plan was as follows: the allocation method was single blind. The order of registration of emergency nurses was numbered. The number of emergency nurses was randomly assigned to the blank, intervention, and control groups using SPSS22.0. The results of randomization assignment are provided in the Supplementary Appendix 1.

This study focused on emergency nurses. The inclusion and exclusion criteria for research subjects were as follows. Inclusion criteria were as follows: (1) holder of a nurse practice certificate, (2) at least 3 years of experience in the emergency department, (3) comprehension of the research purpose and voluntary participation in the study, and (4) sufficient time to participate. Exclusion criterion was as follows: participants who withdrew midway because of health reasons.

Assuming that the test level *α* = 0.05 and the test power 1 − *β* = 0.9, according to the previous pilot experiment, the psychological resilience score of the blank control group was 64.15 ± 11.16, the psychological resilience of the intervention group increased by at least 20%, the sample size of each group should be 18, respectively, plus 15% systematic bias, the sample size of each group was 21, and the total number of cases was 63. The sample size was calculated using MedCalc16.

## 2. Methods

This study was undertaken by a national-level continuing education project applied by the Emergency Department of the West China Hospital of Sichuan University. The training program contained seven teachers who specialized in emergency nursing, eight teachers who specialized in disaster nursing, and five teachers who specialized in psychology. The research-related information was explained 1 h before training and then the participants provided signed informed consent. Relevant information was collected from the three groups before the training using a questionnaire survey method. Afterwards, the three groups were assigned to separate rooms for different types of training.

### 2.1. Educational Program

The 3-day educational program was developed by the researchers following a review of the relevant literature and based on the ICN Framework of Disaster Nursing Competencies 2.0 [17]. In-depth interviews and expert consultation were performed to establish a training framework. A two-round Delphi method was applied, and finally, a training system was established. This topic covered the weak spot of nurses' disaster skills mentioned in the research of Dong et al., such as disaster emergency command systems.

The psychological resilience training was as follows: (1) psychological support and health education ability (1 h); the teacher taught theoretical courses and invited emergency nurses to speak about their own crisis in the disaster situation. Disaster psychological crisis intervention (1 h), vulnerability nursing (1 h), mindfulness-based stress reduction (1 h), and relax by meditating and listening to soothing music under the guidance of teacher; (2) skill drills for disaster psychological construction (1 h), psychological simulation of patients in disaster trauma scenarios (1 h), and training in psychological first-aid skills (1 h). A number of victims had psychological problems at the simulated disaster site, and the emergency nurses provided psychological first aid to the victims. The training period was one day.

The disaster skills training was as follows: (1) module for improving disaster prevention and preparedness capabilities (3 h): sand-table deduction of triage, personal protective equipment, and emergency disposal of hazardous chemical accidents; (2) emergency rescue capability improvement module (3 h): basic trauma life support and process of trauma assessment and treatment; and (3) module for improving resource allocation and management capabilities (3 h): the incident emergency command system (ICS) and simulate how to deal with the rapid increase in casualties. The training period was two days.

Details of the training sessions are available in Supplementary Appendix 2.

#### 2.1.1. Intervention Group

This group was trained in psychological resilience and disaster nursing skills for 3 days.

#### 2.1.2. Control Group

This group underwent 2 days of disaster nursing skills training, consistent with the intervention group. On the third day, no psychological resilience training was performed. The manual concerning the improvement of psychological resilience was distributed to the control group. The manual contained theoretical knowledge in the form of images and texts and was required to be completed within 1 day.

#### 2.1.3. Blank Group

This group received no intervention.

Three days later, after the data collection, the participants in the control and blank groups took part in the educational program. The evaluation index was psychological resilience and disaster nursing ability levels.

The evaluation tool examined the following: (1) the general information questionnaire was based on a literature review, which primarily includes the participants' general social demographic data and disaster relief experience (Supplementary Appendix 3). (2) The Connor–Davidson Resiliency Scale (C-D RS) [18] (Supplementary Appendix 4), which is primarily used to measure the psychological adaptation level of individuals, included 3 dimensions (tenacity, strength, and optimism) and 25 items. C-D RS uses a 5-point Likert-type scale, from 0 to 4 points, with the total score ranging from 0 to 100 points. The higher the total score, the higher the psychological resilience level. Researchers in [19] adopted the Chinese-adapted scale for measurement. Cronbach's *α* coefficient of the scale was 0.840, and Cronbach's *α* coefficient of each dimension was 0.889, 0.838, and 0.762. (3) The Nurses' Disaster Nursing Ability Assessment Scale is a nurse's disaster nursing ability measurement tool compiled by Heng et al. [17] (Supplementary Appendix 5). It is based on the ICN disaster nursing ability framework, including 9 dimensions and 55 items in total. A 5-point Likert scoring method was used, from 1 to 5 points, ranging from “very poorly done” to “very well done.” The score ranged from 55 to 275 points, and the higher the total score, the stronger the disaster nursing ability. Cronbach's *α* coefficient of the scale was 0.974, the test-retest reliability coefficient was 0.999, the content validity index was 0.93, and the correlation coefficient between each first-level index and the total was between 0.805 and 0.951.

### 2.2. Statistical Analysis

The data collected in the study were analyzed using SPSS 22.0 statistical package software. Comparisons were performed to evaluate the descriptive characteristics, disaster nursing ability, and psychological resilience levels of the participants in the blank, intervention, and control groups. Frequency counts and percentages were used to analyze the data of emergency nurses, such as gender and ethnicity. The mean and standard deviation were used to describe the measurement data such as the score of each questionnaire. Analysis of variance was used to compare the quantitative data between the groups. The chi-square test was used to compare the classified data between groups. A two-tailed test was used for all *P* values, and the test level was *α* = 0.05.

## 3. Findings

A total of 93 emergency nurses participated in this study: 34 in the blank group, 31 in the intervention group, and 28 in the control group. There were no statistical differences among the three groups concerning general information, e.g., experiencing disaster events, being involved in disaster rescue, and participating in disaster-related training (*P* > 0.05), as presented in Table 1.

The average total score of resilience of the three groups in the pretest was 64.15 ± 11.16, 64.15 ± 11.16, and 65.14 ± 5.80. There was no significant difference between the three groups in the pretest (*P* > 0.05). The average total score of resilience of the three groups in the posttest was 61.76 ± 9.24, 75.26 ± 11.15, and 75.26 ± 11.15. The difference in resilience scores among all the groups in the posttest was statistically significant (*P* < 0.05), as presented in Table 2.

The average total score of disaster nursing ability of the three groups in the pretest was 196.15 ± 22.29, 194.13 ± 25.63, and 197.07 ± 30.64. There was no statistically significant difference in the disaster nursing ability score of each group (*P* > 0.05). In the posttest, the average total score of disaster nursing ability of the three groups was 194.29 ± 26.82, 224.52 ± 32.77, and 229.07 ± 30.64. The difference in disaster nursing ability scores between the groups was statistically significant (*P* < 0.05). The scores of each dimension of disaster nursing ability of the three groups are presented in Table 2.

The scores of psychological resilience and disaster nursing ability of the three groups in the pretest and posttest were compared using a paired-sample *t*-test. The results were as follows: in the blank group, there was no significant difference in the total score of resilience (strength and tenacity dimensions) in the pretest and posttest (*P* > 0.05). There was no significant difference in the total score of disaster nursing ability and three dimensions of disaster preparedness ability, response ability, and recovery and reconstruction ability in the pretest and posttest (*P* > 0.05). The specific results are presented in Table 3.

In the intervention group, the psychological resilience, strength and optimism dimensions, total disaster nursing ability, and scores of each dimension in the posttest were higher than those in the pretest. The differences were statistically significant (*P* < 0.05). The specific results are presented in Table 4.

In the control group, psychological resilience and the average score of each dimension in the posttest were higher than those in the pretest, but there was no statistically significant difference (*P* > 0.05). The total score and the score of each dimension of disaster nursing ability in the posttest were higher than psychological resilience and the average score of each dimension in the pretest, and the differences were statistically significant (*P* < 0.05). The specific results are presented in Table 5.

## 4. Discourse

In the pretest, the average score of psychological resilience of emergency nurses was 65.66 ± 1.10, which was at the medium level, consistent with the results of other researchers [12]. Under a public health emergency, the surge of patients inevitably induced increased workload, prolonged procedures, and fatigue, which all challenged the physical tolerability of emergency department nurses, and subsequently difficulties in adapting with the busy schedule and reduced resilience [20]. The study which was conducted during the COVID-19 pandemic shows that resilience among emergency nurses is indeed low. In the pretest, the total score of disaster nursing ability of emergency nurses was 202.90 ± 2.59, which was in the upper-middle level and higher than the intensive care nurse in Jinan, China [15]. This is because emergency nurses have more experience in rescuing large groups of wounded patients and, hence, deal with more critical patients during their usual work. In addition, emergency nurses have advantages in disaster nursing because they participate in emergency rescue work [21]. Among the 93 nurses included in this study, 67% had experienced disaster events, 36.56% had participated in disaster on-site rescue, and 48.39% had participated in disaster-related training, indicating that emergency nurses had participated in disaster nursing-related training and had experienced disaster response and rescue.

In recent years, disaster nursing education has gradually increased [22]. The accumulation of disaster rescue experience, formal training, and emergency training contributes to the improvement of nurses' disaster nursing ability [23].

In this study, both the intervention and control groups received disaster nursing knowledge and skills training. The scores of the two groups were significantly higher than in the pretest, indicating that training can improve the disaster skills of nurses [24]. The United States began to provide disaster preparedness education for nurses at 2001, while disaster education for nurses in China began after the Wenchuan Earthquake in 2008 [25]. In the blank group, which received no training, there was no significant difference in the competence of disaster nursing in the pretest and posttest, indicating that the experience of disaster training is an important factor influencing disaster nursing ability. It is necessary to strengthen the disaster nursing ability training for emergency nurses.

Through the implementation of psychological resilience-related training, the psychological resilience scores of the intervention group were significantly improved (*P* < 0.05), and this is consistent with the results of Martin C Delane's and Wen Jiao Huang's study [26, 27]. However, there was no statistically significant difference in the psychological resilience score of the control group in the pretest and posttest (*P* > 0.05). The psychological resilience improvement course constructed by the research team was effective in enhancing psychological resilience. Psychological knowledge and skills training can cultivate subjective well-being and the optimistic personality of nurses to improve the level of psychological resilience [28]. The resilience scores of the intervention group were also significantly higher than those of the control group.

Psychological resilience was positively correlated with disaster nursing ability [13]. Psychological first-aid (PFA) training has been found to be effective at increasing people's confidence in engaging in rescue activities, in turn enhancing the resilience of rescuers [29, 30]. The psychological knowledge of disaster nursing ability can improve disaster rescue ability.

The innovation of this study was that through the psychological simulation of patients in disaster trauma scenes and the skill exercise of psychological first-aid ability training, nurses became more confident in dealing with the difficulties encountered in disaster rescue, more optimistic about disaster events, and more capable of handling disaster events. This is one of the best interventions to improve nurses' resilience.

### 4.1. Limitations

In this study, the intervention of psychological resilience did not significantly improve the tenacity dimension and the research center still needs to perform follow-ups to further explore the methods of psychological resilience intervention, such as the adoption of mindfulness-based stress reduction [31] and cognitive behavioral group psychological counseling [32]. Furthermore, the number of participants was not sufficient, and because of time constraints, a third survey was not conducted for a longer period after the end of the study. Therefore, it is not possible to understand the long-term impact of the study on the psychological resilience of nurses.

## 5. Conclusion

In this study, we designed a program aimed at enhancing the psychological resilience of emergency nurses in dealing with disasters. Simultaneously training psychological resilience knowledge along with disaster response skills resulted in a more pronounced improvement in the capabilities of emergency nurses.

## Figures and Tables

**Table 1:** General demographic data of the subjects.

Categories		Blank group (*N* = 34)	Intervention group (*N* = 31)	Control group (*N* = 28)	*χ* ^2^	*P* value
Gender	Male	7	5	4	0.455	0.792
	Female	27	26	24		

Educational background	Junior college	13	7	6	2.821	0.244
	Undergraduate course	21	24	22		

Professional title	Nurse	2	6	7	8.296	0.217
	Senior nurse	23	15	12		
	Supervisor nurse	9	10	8		
	Associate professor of nursing	0	0	1		

Have you ever experienced a disaster event?	Yes	23	20	20	0.322	0.851
	No	11	11	8		

Have you ever participated in disaster relief?	Yes	14	9	11	1.160	0.560
	No	20	22	17		

Have you participated in disaster-related training?	Yes	13	19	13	3.513	0.173
	No	21	12	15		

**Table 2:** Scores of psychological ability and disaster nursing ability of the three groups of subjects before and after intervention (*N* = 93).

Categories		Blank group (*N* = 34)	Intervention group (*N* = 31)	Control group (*N* = 28)	*χ* ^2^	*P* value
Gender	Male	7	5	4	0.455	0.792
	Female	27	26	24		

Educational background	Junior college	13	7	6	2.821	0.244
	Undergraduate course	21	24	22		

Professional title	Nurse	2	6	7	8.296	0.217
	Senior nurse	23	15	12		
	Supervisor nurse	9	10	8		
	Associate professor of nursing	0	0	1		

Have you ever experienced a disaster event?	Yes	23	20	20	0.322	0.851
	No	11	11	8		

Have you ever participated in disaster relief?	Yes	14	9	11	1.160	0.560
	No	20	22	17		

Have you participated in disaster-related training?	Yes	13	19	13	3.513	0.173
	No	21	12	15		

**Table 3:** Comparison of results before and after training of emergency nurses in the blank control group (*N* = 34).

Subjects	Blank group		*t*	*P* value
	Pretest	Posttest		
Resilience	64.15 ± 11.16	61.76 ± 9.24	1.873	0.070
Tenacity	33.03 ± 6.04	32.82 ± 4.77	0.257	0.789
Strength	19.03 ± 4.21	18.65 ± 3.39	0.725	0.474
Optimism	12.09 ± 1.85	10.29 ± 1.98	5.124	<0.001
Disaster nursing ability	196.15 ± 22.29	194.29 ± 26.82	0.474	0.639
Capacity in disaster reduction and prevention	25.09 ± 5.27	25.91 ± 5.34	−1.102	0.279
Disaster preparedness	73.91 ± 8.97	72.15 ± 10.34	0.969	0.339
Coping capacity	79.24 ± 8.60	79.18 ± 11.35	0.031	0.976
Recovery and reconstruction capacity	17.91 ± 3.04	17.06 ± 3.05	1.867	0.071

**Table 4:** Comparison of results of emergency nurses before and after training in the intervention group (*N* = 31).

Subjects	Intervention group		*t*	*P* value
	Pretest	Posttest		
Resilience	64.74 ± 11.44	75.26 ± 11.15	−6.054	<0.001
Tenacity	33.19 ± 6.78	37.45 ± 7.53	−3.591	0.001
Strength	19.35 ± 3.71	23.19 ± 4.67	−5.387	<0.001
Optimism	12.19 ± 2.30	14.61 ± 2.38	−3.729	0.001
Disaster nursing ability	194.13 ± 25.63	224.52 ± 32.77	−5.467	<0.001
Capacity in disaster reduction and prevention	28.48 ± 6.07	31.42 ± 5.12	−2.758	0.010
Disaster preparedness	70.71 ± 9.93	83.10 ± 11.94	−5.185	<0.001
Coping capacity	76.74 ± 11.11	89.87 ± 13.34	−5.238	<0.001
Recovery and reconstruction capacity	18.19 ± 3.97	20.13 ± 3.26	−2.915	0.007

**Table 5:** Comparison of results before and after training of emergency nurses in the control group (*N* = 28).

Subjects	Control group		*t*	*P* value
	Pretest	Posttest		
Resilience	65.14 ± 5.80	68.29 ± 7.25	−1.929	0.064
Tenacity	34.71 ± 3.42	36.61 ± 5.52	−1.570	0.128
Strength	18.50 ± 2.50	19.00 ± 1.59	−0.841	0.408
Optimism	11.93 ± 2.32	12.68 ± 2.34	−1.523	0.139
Disaster nursing ability	197.07 ± 30.64	229.07 ± 30.64	−5.971	<0.001
Capacity in disaster reduction and prevention	28.96 ± 4.72	32.61 ± 4.91	−3.632	0.001
Disaster preparedness	71.00 ± 9.17	85.14 ± 10.33	−5.601	0.001
Coping capacity	77.57 ± 6.82	90.64 ± 14.19	−4.581	<0.001
Recovery and reconstruction capacity	19.54 ± 2.89	20.68 ± 3.38	−2.192	0.037

## Data Availability

The data used to support the findings of this study are included within the article.

## Supplementary Materials

Supplementary Materials Supplementary 1. Supplementary Appendix 1: the results of randomization assignment. Supplementary 2. Supplementary Appendix 2: details of the training sessions. Supplementary 3. Supplementary Appendix 3: the general information questionnaire. Supplementary 4. Supplementary Appendix 4: the Connor–Davidson Resiliency Scale (C-D RS). Supplementary 5. Supplementary Appendix 5: the Nurses' Disaster Nursing Ability Assessment Scale. Supplementary 6. Table 1: general demographic data of the subjects. Supplementary 7. Table 2: scores of psychological ability and disaster nursing ability of the three groups of subjects before and after intervention (*N* = 93). Supplementary 8. Table 3: comparison of results before and after the training of emergency nurses in blank control group (*N* = 34). Supplementary 9. Table 4: comparison of results of emergency nurses before and after training in the intervention group (*N* = 31). Supplementary 10. Table 5: comparison of results before and after training of emergency nurses in the control group (*N* = 28).

## References

[1] Varghese A., George G., Kondaguli S. V., Naser A. Y., Khakha D. C., Chatterji R. (2021). Decline in the mental health of nurses across the globe during COVID-19: a systematic review and meta-analysis. *Journal of Global Health*.

[2] Miglani M., Upadhyay P., Mahajan R. (2022). Psychological resilience, coping, and distress in admitted patients with COVID-19 infection. *The Primary Care Companion for Central nervous system Disorders*.

[3] Sisto A., Vicinanza F., Campanozzi L. L., Ricci G., Tartaglini D., Tambone V. (2019). Towards a transversal definition of psychological resilience: a literature review. *Medicina*.

[4] Barbarin O. A. (1987). Psychosocial risks and invulnerability: a review of the theoretical and empirical bases of preventive family-focused services for survivors of childhood cancer. *Journal of Psychosocial Oncology*.

[5] Luthar S. S., Cicchetti D., Becker B. (2000). The construct of resilience: a critical evaluation and guidelines for future work. *Child Development*.

[6] Cooper A. L., Brown J. A., Rees C. S., Leslie G. D. (2020). Nurse resilience: a concept analysis. *International Journal of Mental Health Nursing*.

[7] Labrague L. J. (2021). Psychological resilience, coping behaviours and social support among health care workers during the COVID-19 pandemic: a systematic review of quantitative studies. *Journal of Nursing Management*.

[8] Wei H., Roberts P., Strickler J., Corbett R. W. (2019). Nurse leaders’ strategies to foster nurse resilience. *Journal of Nursing Management*.

[9] Hart P. L., Brannan J. D., De Chesnay M. (2014). Resilience in nurses: an integrative review. *Journal of Nursing Management*.

[10] Kunzler A. M., Helmreich I., Chmitorz A. (2020). Psychological interventions to foster resilience in healthcare professionals. *Cochrane Database of Systematic Reviews*.

[11] Foster K., Cuzzillo C., Furness T. (2018). Strengthening mental health nurses’ resilience through a workplace resilience programme: a qualitative inquiry. *Journal of Psychiatric and Mental Health Nursing*.

[12] Sánchez-Zaballos M., Mosteiro-Díaz M. P. (2021). Resilience among professional health workers in emergency services. *Journal of Emergency Nursing*.

[13] Mao X., Fung O. W., Hu X., Loke A. Y. (2022). Characteristics of resilience among disaster rescue workers: a systematic review. *Disaster Medicine and Public Health Preparedness*.

[14] International Council of Nurses (2019). Core competencies in disaster nursing. https://www.icn.ch/sites/default/files/inline-files/ICN_Disaster-Comp-Report_WEB.pdf.

[15] Jiang M., Sun M., Zhang X., Luan X. R., Li R. J. (2022). Disaster nursing competency of intensive care nurses in Jinan, China: a multicenter cross-sectional study. *Journal of Nursing Research*.

[16] McNeill C., Adams L., Heagele T., Swanson M., Alfred D. (2020). Emergency preparedness competencies among nurses: implications for nurse administrators. *The Journal of Nursing Administration*.

[17] Heng W., Yijuan C., Xiuying H. (2020). Psychometric testing of the nurse’ disaster nursing competency evaluation tool. *Chinese Nursing Management*.

[18] Connor K. M., Davidson J. R. (2003). Development of a new resilience scale: the connor-davidson resilience scale (CD-RISC). *Depression and Anxiety*.

[19] Cheng C., Dong D., He J., Zhong X., Yao S. (2020). Psychometric properties of the 10-item Connor-Davidson Resilience Scale (CD-RISC-10) in Chinese undergraduates and depressive patients. *Journal of Affective Disorders*.

[20] Lu J., Xu P., Ge J., Zeng H., Liu W., Tang P. (2023). Analysis of factors affecting psychological resilience of emergency room nurses under public health emergencies. *Inquiry*.

[21] Yang J., Kim K. H. (2022). Effect of the strategic thinking, problem solving skills, and grit on the disaster triage ability of emergency room nurses. *International Journal of Environmental Research and Public Health*.

[22] Geng C., Luo Y., Pei X., Chen X. (2021). Simulation in disaster nursing education: a scoping review. *Nurse Education Today*.

[23] Jose M. M., Dufrene C. (2014). Educational competencies and technologies for disaster preparedness in undergraduate nursing education: an integrative review. *Nurse Education Today*.

[24] Fil S. L., Champion J. D., Christiansen B. (2021). Perceptions of disaster management knowledge and skills among advanced practice registered nurses. *Journal of the American Association of Nurse Practitioners*.

[25] Littleton-Kearney M. T., Slepski L. A. (2008). Directions for disaster nursing education in the United States. *Critical Care Nursing Clinics of North America*.

[26] Delaney M. C. (2018). Caring for the caregivers: evaluation of the effect of an eight-week pilot mindful self-compassion (MSC) training program on nurses’ compassion fatigue and resilience. *PLoS One*.

[27] Huang W., Li L., Zhuo Y., Zhang J. (2022). Analysis of resilience, coping style, anxiety, and depression among rescue nurses on EMTs during the disaster preparedness stage in Sichuan, China: a descriptive cross-sectional survey. *Disaster Medicine and Public Health Preparedness*.

[28] Pollock A., Campbell P., Cheyne J. (2020). Interventions to support the resilience and mental health of frontline health and social care professionals during and after a disease outbreak, epidemic or pandemic: a mixed methods systematic review. *Cochrane Database of Systematic Reviews*.

[29] Everly G. S., Barnett D. J., Sperry N. L., Links J. M. (2010). The use of psychological first aid (PFA) training among nurses to enhance population resiliency. *International Journal of Emergency Mental Health*.

[30] Allen B., Brymer M. J., Steinberg A. M. (2010). Perceptions of psychological first aid among providers responding to Hurricanes Gustav and Ike. *Journal of Traumatic Stress*.

[31] Janssen M., Heerkens Y., Kuijer W., van der Heijden B., Engels J. (2018). Effects of Mindfulness-Based Stress Reduction on employees’ mental health: a systematic review. *PLoS One*.

[32] Demir S., Ercan F. (2022). The effectiveness of cognitive behavioral therapy-based group counseling on depressive symptomatology, anxiety levels, automatic thoughts, and coping ways Turkish nursing students: a randomized controlled trial. *Perspectives in Psychiatric Care*.

