# Supplementary material for: Enhancing Emergency Nurses' Disaster Nursing Ability and Psychological Resilience: A Randomized Controlled Trial
Source: Emerg Med Int. 2023 Nov 27;2023:6108057. doi: 10.1155/2023/6108057 (PMC10695688; doi:10.1155/2023/6108057)
Supplement: Supplementary Materials — Supplementary 1. Supplementary Appendix 1: the results of randomization assignment. Supplementary 2. Supplementary Appendix 2: details of the training sessions. Supplementary 3. Supplementary Appendix 3: the general information questionnaire. Supplementary 4. Supplementary Appendix 4: the Connor–Davidson Resiliency Scale (C-D RS). Supplementary 5. Supplementary Appendix 5: the Nurses' Disaster Nursing Ability Assessment Scale. Supplementary 6. Table 1: general demographic data of the subjects. Supplementary 7. Table 2: scores of psychological ability and disaster nursing ability of the three groups of subjects before and after intervention (N = 93). Supplementary 8. Table 3: comparison of results before and after the training of emergency nurses in blank control group (N = 34). Supplementary 9. Table 4: comparison of results of emergency nurses before and after training in the intervention group (N = 31). Supplementary 10. Table 5: comparison of results before and after training of emergency nurses in the control group (N = 28). [file 6108057.f1.zip › Supplementary Appendix 2.pdf]

“Construction and application e

Theoretical basis of curriculum: The disaster nursing co
aspects: disaster reduction/prevention ability, disaster
integrated the core competence of disaster nursing and r
rational allocation and management ability of resources,
scholars in our country.

time	topic
	pre-test
8:15-8:30	Registration
8:30-8:45	Introduction about the Course
9:00-10:00	Overview of disaster trauma care
10:00-11:00	Disaster site assessment
11:00-12:00	Mass casualty incident handling
12:00-13:00	lunch
13:00-14:00	An overview of the Incident Emergency Command System (ICS)
14:00-15:00	Emergency plan and exercise for public health emergencies
15:00-16:00	Emergency handling and personal protection of hazardous chemical accidents
	the course summary

9:00-10:00	workshop one,	Skill 1: Locking and spinal restraint technique
10:00-11:00		Skill 2: Wound hemostasis, bandaging and fixation technique
11:00-12:00		Skill 3: Airway management
12:00-13:00	lunch	
13:00-14:00	workshop two	Skill 4: Personal protective equipment application
14:00-15:00		Skill 5: ICS desktop deduction
	the course summary	
09:00-12:00	Disaster psychological crisis intervention and vulnerable population care	

12:00-13:00	lunch	
13:00-16:30	workshop three	Skill 6: Disaster psychological construction

ffect of nurses’ disaster rescue ability training s
psychological resilience

ɔre competency framework for nurses formulated by the International Council of Nurses
· preparedness ability, response ability, and recovery/rehabilitation ability. Chines
·efined it into four modules: disaster prevention and preparedness ability, emergency
psychological support and health education ability. This course is based on the coı

content
first day
Disaster-related terminology, disaster preparedness and preparedness kits, research status
Disaster, injury, resources, risk
Trauma classification (SALT.START), casualty evacuation and evacuation, hospital disposal management, etc
ICS command system framework, the role of various elements, ICS operation, disaster site command, hospital casualty response
Emergency plan overview, plan preparation, drill design
Types of hazardous chemicals and hazardous chemical accidents, emergency treatment, personal protective tools and use; Understand the signs and symptoms of human exposure to chemical, biological, radiological, explosive, and nuclear substances, and identify potential outbreaks of disease

The second day
Basic trauma life support: dressing, hemostasis, fixation and transport
Endotracheal intubation, difficult airway, oropharyngeal airway, nasopharyngeal airway, cricothyroid membrane puncture, combined esophagotracheal tube, laryngeal mask airway, etc
Introduction and training of personal protective equipment
ICS composition, division of labor, ICS operation and other desktop deduction
pro-test
third day
Help distinguish between adaptive and non-adaptive responses to disasters, and guide the correct face of psychological crises, skills, responsibilities, and ethics

To simulate the psychology of patients in various disaster trauma scenes and train psychological first aid

pro-test

ystem based on

s (ICN) includes four<br>se scholars have highly<br>y rescue ability,<br>re competence refined by	

teacher	period (min)
	15
	15
Lei Ye	60
Yu Zhuo	60
Zhenfei Yuan	60
Wenjiao Huan	60
Shiyuan Tang	60
Jing Zhao	60

Wei Wang/Yue	60
Jiale Tong	60
Zhenfei Yuan	60
Jing Zhao/Li	60
Wenjiao Huan	60
Xiandong Meng	180

Yu Zhuo/Jing	180
