# Supplementary material for: Enhancing Emergency Nurses' Disaster Nursing Ability and Psychological Resilience: A Randomized Controlled Trial
Source: Emerg Med Int. 2023 Nov 27;2023:6108057. doi: 10.1155/2023/6108057 (PMC10695688; doi:10.1155/2023/6108057)
Supplement: Supplementary Materials — Supplementary 1. Supplementary Appendix 1: the results of randomization assignment. Supplementary 2. Supplementary Appendix 2: details of the training sessions. Supplementary 3. Supplementary Appendix 3: the general information questionnaire. Supplementary 4. Supplementary Appendix 4: the Connor–Davidson Resiliency Scale (C-D RS). Supplementary 5. Supplementary Appendix 5: the Nurses' Disaster Nursing Ability Assessment Scale. Supplementary 6. Table 1: general demographic data of the subjects. Supplementary 7. Table 2: scores of psychological ability and disaster nursing ability of the three groups of subjects before and after intervention (N = 93). Supplementary 8. Table 3: comparison of results before and after the training of emergency nurses in blank control group (N = 34). Supplementary 9. Table 4: comparison of results of emergency nurses before and after training in the intervention group (N = 31). Supplementary 10. Table 5: comparison of results before and after training of emergency nurses in the control group (N = 28). [file 6108057.f1.zip › Supplementary Appendix 3.docx]

The general information questionnaire

1. Your gender：□male □female
2. your age:___________ years old
3. Years of nursing work:___________ year
4. Years of experience in emergency department:___________ year
5. your job title：□nurse □Senior nurse □supervisor nurse  □co-chief superintendent nurse □chief superintendent nurse
6. Your education background： □technical secondary school
7. □junior college □undergraduate course □Master's degree or above
8. Your marital status：□discoverture □married □divorced □bereft of one's spouse
9. Do you have any children：□yes □no_
10. The type of your work contract：□be on regular payroll □contract □human agency □else___________
11. The status of your work：
12. □original unit □Have been sent to Hubei to support □During quarantine □else___________
13. Have you worked with patients who are confirmed or suspected to have the novel coronavirus：□yes □no
14. Do you have any disaster relief experience (excluding this outbreak)：□yes □no
15. You have participated in several disaster relief operations：___________ times
16. Among them, how many participated in the disaster scene rescue ：___________
17. Among them, how many participated in hospital rescue (rear rescue) several times：___________
