# Supplementary material for: Enhancing Emergency Nurses' Disaster Nursing Ability and Psychological Resilience: A Randomized Controlled Trial
Source: Emerg Med Int. 2023 Nov 27;2023:6108057. doi: 10.1155/2023/6108057 (PMC10695688; doi:10.1155/2023/6108057)
Supplement: Supplementary Materials — Supplementary 1. Supplementary Appendix 1: the results of randomization assignment. Supplementary 2. Supplementary Appendix 2: details of the training sessions. Supplementary 3. Supplementary Appendix 3: the general information questionnaire. Supplementary 4. Supplementary Appendix 4: the Connor–Davidson Resiliency Scale (C-D RS). Supplementary 5. Supplementary Appendix 5: the Nurses' Disaster Nursing Ability Assessment Scale. Supplementary 6. Table 1: general demographic data of the subjects. Supplementary 7. Table 2: scores of psychological ability and disaster nursing ability of the three groups of subjects before and after intervention (N = 93). Supplementary 8. Table 3: comparison of results before and after the training of emergency nurses in blank control group (N = 34). Supplementary 9. Table 4: comparison of results of emergency nurses before and after training in the intervention group (N = 31). Supplementary 10. Table 5: comparison of results before and after training of emergency nurses in the control group (N = 28). [file 6108057.f1.zip › Supplementary Appendix 4.docx]

## CD-RISC Chinese version of the psychological Resilience Scale

The scale was developed by American psychologists Connor and Davidson. It is mainly used to measure the level of individual psychological adaptation, which contains 25 items and 3 dimensions, including strength dimension (contains 8 items), optimism dimension (contains 4 items), tenacity and control dimension (contains 13 items), using Likert5 scale, "never like this" is 0 points, "rarely like this" is 1 points, "sometimes like this" is 2 points. "This is often the case" was given a score of 3 and "almost always" a score of 4 out of a total of 100, with higher scores indicating better psychological adaptability. The Cronbach's α coefficient of the scale was 0.840, and the Cronbach's α coefficients of the three dimensions of tenacity, self-reliance and optimism were 0.889, 0.838 and 0.762, respectively

| content | never like this(0) | rarely like this(1) | sometimes like this(2) | This is often the case(3) | almost always(4) |
| --- | --- | --- | --- | --- | --- |
| 1.I can adapt to change |  |  |  |  |  |
| 2. I have close and strong relationships |  |  |  |  |  |
| 3. I sometimes turn to God or fate |  |  |  |  |  |
| 4. I can handle anything unexpected |  |  |  |  |  |
| 5. The success of the past gives me the confidence to overcome new challenges |  |  |  |  |  |
| 6. I can see the funny side of things |  |  |  |  |  |
| 7. I can handle increasing pressure |  |  |  |  |  |
| 8. I can bounce back quickly after an illness or difficulty |  |  |  |  |  |
| 9. I think everything happens for a reason |  |  |  |  |  |
| 10. I did my best anyway, you know |  |  |  |  |  |
| 11. I believe I can achieve my goals |  |  |  |  |  |
| 12. Although I consider things hopeless, I will not give up |  |  |  |  |  |
| 13. I know how to ask for help when I'm in trouble |  |  |  |  |  |
| 14. I can concentrate and think under pressure |  |  |  |  |  |
| 15. I like to be in the driver's seat when solving problems |  |  |  |  |  |
| 16. I will never be discouraged in the face of failure |  |  |  |  |  |
| 17. I consider myself a strong person |  |  |  |  |  |
| 18. I'm decisive |  |  |  |  |  |
| 19. I can deal with unpleasant feelings |  |  |  |  |  |
| 20. I have to go with my gut sometimes |  |  |  |  |  |
| 21. When I do things, I have a clear goal |  |  |  |  |  |
| 22. I feel like I'm in control of my life |  |  |  |  |  |
| 23.I like challenging |  |  |  |  |  |
| 24.I will try to reach my goal |  |  |  |  |  |
| 25.I will be proud of my achievements |  |  |  |  |  |
