# Supplementary material for: Enhancing Emergency Nurses' Disaster Nursing Ability and Psychological Resilience: A Randomized Controlled Trial
Source: Emerg Med Int. 2023 Nov 27;2023:6108057. doi: 10.1155/2023/6108057 (PMC10695688; doi:10.1155/2023/6108057)
Supplement: Supplementary Materials — Supplementary 1. Supplementary Appendix 1: the results of randomization assignment. Supplementary 2. Supplementary Appendix 2: details of the training sessions. Supplementary 3. Supplementary Appendix 3: the general information questionnaire. Supplementary 4. Supplementary Appendix 4: the Connor–Davidson Resiliency Scale (C-D RS). Supplementary 5. Supplementary Appendix 5: the Nurses' Disaster Nursing Ability Assessment Scale. Supplementary 6. Table 1: general demographic data of the subjects. Supplementary 7. Table 2: scores of psychological ability and disaster nursing ability of the three groups of subjects before and after intervention (N = 93). Supplementary 8. Table 3: comparison of results before and after the training of emergency nurses in blank control group (N = 34). Supplementary 9. Table 4: comparison of results of emergency nurses before and after training in the intervention group (N = 31). Supplementary 10. Table 5: comparison of results before and after training of emergency nurses in the control group (N = 28). [file 6108057.f1.zip › Supplementary Appendix 5.docx]

Disaster nursing ability assessment scale for nurses

Wang Heng and Hu Xiuying took the disaster nursing ability model of the International Council of Nurses (ICN) as the guidance, adopted the Delphi method, and carried out sufficient "localization debugging" based on the disaster nursing ability proposed by the International Council Nurses (ICN). According to the basic national conditions of China and the current situation of clinical nurses' disaster nursing ability, various indicators were strictly screened and modified. Finally, a reliable, comprehensive and accurate assessment tool for clinical nurses' disaster nursing ability was developed, including 4 fields, 9 dimensions and 55 items, such as disaster reduction/prevention, disaster preparedness, response and recovery/reconstruction. The assessment tool is comprehensive and representative. It can be used as an evaluation tool for the disaster nursing ability of clinical nurses in China and an evaluation standard for the training needs of disaster nursing ability of clinical nurses in China. At the same time, it is convenient to compare the disaster nursing ability of nurses in other countries or regions.

| content | degree（5means very good→1means very poor） | | | | |
| --- | --- | --- | --- | --- | --- |
|  | 5 | 4 | 3 | 2 | 1 |
| 1. Risk assessment, identify existing health problems in the workplace, health care resources, etc |  |  |  |  |  |
| 1. Work with multidisciplinary teams to develop measures for the assessment |  |  |  |  |  |
| 1. Understand the principles of isolation, quarantine, purification and discharge of infectious diseases |  |  |  |  |  |
| 1. Participate in disaster preparedness education in the community |  |  |  |  |  |
| 1. Understand the tasks of nurses at different stages of disaster |  |  |  |  |  |
| 1. Clarify the role of governments and organizations in disasters |  |  |  |  |  |
| 1. Participate in the development of disaster plans in your workplace |  |  |  |  |  |
| 1. Identify individual roles in disaster planning and be able to describe them to other members of the team |  |  |  |  |  |
| 1. Attach importance to ethics and protect individual rights, values and dignity |  |  |  |  |  |
| 1. Be aware of potential ethical conflicts and plan for emergencies |  |  |  |  |  |
| 1. Understand relevant laws and regulations and comply with relevant requirements such as security, confidentiality, evidence retention, etc |  |  |  |  |  |
| 1. Be responsible for your own actions in nursing activities |  |  |  |  |  |
| 1. Take good personal safety protection |  |  |  |  |  |
| 1. Identify their own knowledge, skills deficiencies, and take appropriate measures |  |  |  |  |  |
| 1. Be familiar with communication principles and be able to use different communication methods or tools for different groups |  |  |  |  |  |
| 1. Understand the information transfer process in disasters |  |  |  |  |  |
| 1. Be able to find important information in time and report to the appropriate superiors |  |  |  |  |  |
| 1. Focus on information communication with multidisciplinary teams, maintain the consistency of information, and clarify the role of nurses in communication with outside groups such as the media |  |  |  |  |  |
| 1. Ability to keep relevant records and documents and submit reports as required |  |  |  |  |  |
| 1. Familiar with disaster-related terminology |  |  |  |  |  |
| 1. Master first-aid knowledge and skills (such as cardiopulmonary resuscitation, debridement and hemostasis, bandaging and fixing, etc.) |  |  |  |  |  |
| 1. Participate in workplace disaster drills |  |  |  |  |  |
| 1. Update the new ideas and knowledge of disaster care in a timely manner |  |  |  |  |  |
| 1. Promote disaster-related research |  |  |  |  |  |
| 1. Attend necessary disaster care training |  |  |  |  |  |
| 1. Develop individual and family disaster preparedness plans |  |  |  |  |  |
| 1. Clarify the role of nurses in different disaster relief sites |  |  |  |  |  |
| 1. Prepare a personal disaster preparedness package |  |  |  |  |  |
| 1. Can quickly assess the disaster situation |  |  |  |  |  |
| 1. Can quickly collect health history, identify nursing needs, use critical thinking to propose nursing plans, and take corresponding measures |  |  |  |  |  |
| 1. Be able to recognize the occurrence and symptoms of infectious diseases and take active measures |  |  |  |  |  |
| 1. Understand the signs and symptoms of human exposure to chemical, biological, radiological, explosive, and nuclear substances, and identify potential outbreaks of disease |  |  |  |  |  |
| 1. Be able to judge the demand for purification, isolation, quarantine and other measures, and take appropriate treatment measures |  |  |  |  |  |
| 1. Triage |  |  |  |  |  |
| 1. Create a safe care environment |  |  |  |  |  |
| 1. Can provide safe transit |  |  |  |  |  |
| 1. Can carry out safety management of drugs, vaccines, etc |  |  |  |  |  |
| 1. Effective infection control measures can be taken to prevent the spread of disease |  |  |  |  |  |
| 1. The effectiveness of nursing measures can be evaluated and improved |  |  |  |  |  |
| 1. Record the nursing process in time |  |  |  |  |  |
| 1. Work with other agencies to help survivors get in touch with their families |  |  |  |  |  |
| 1. Understand the stages of psychological response and possible behavioral responses to disasters |  |  |  |  |  |
| 1. To understand the psychological impact of disasters on different populations and provide appropriate interventions |  |  |  |  |  |
| 1. It can effectively establish therapeutic nurse-patient relationship |  |  |  |  |  |
| 1. It can distinguish between adaptive and non-adaptive responses to disasters |  |  |  |  |  |
| 1. Provide appropriate psychological support and referrals for survivors and rescuers who require intensive psychological care |  |  |  |  |  |
| 1. Vulnerable populations identified as at risk (e.g. chronically ill, elderly, children, pregnant women, persons with disabilities, etc.) |  |  |  |  |  |
| 1. Ensure that disaster plans meet the needs of vulnerable populations |  |  |  |  |  |
| 1. Understands, identifies and meets the care needs of vulnerable people wherever possible |  |  |  |  |  |
| 1. Ability to work with multidisciplinary teams to provide continuity of care programs for vulnerable populations |  |  |  |  |  |
| 1. Can develop care plan to meet the short and long term physical and psychological care needs of survivors |  |  |  |  |  |
| 1. Be able to detect changes in survivors' care needs in time and make timely revisions |  |  |  |  |  |
| 1. Refer survivors with special needs to other organizations |  |  |  |  |  |
| 1. Can guide survivors to prevent illness or injury after treatment |  |  |  |  |  |
| 1. Sharing information on resources in disaster care |  |  |  |  |  |
