# Supplementary figures and images for: Enhancing Emergency Nurses' Disaster Nursing Ability and Psychological Resilience: A Randomized Controlled Trial
Source: Emerg Med Int. 2023 Nov 27;2023:6108057. doi: 10.1155/2023/6108057 (PMC10695688; doi:10.1155/2023/6108057)

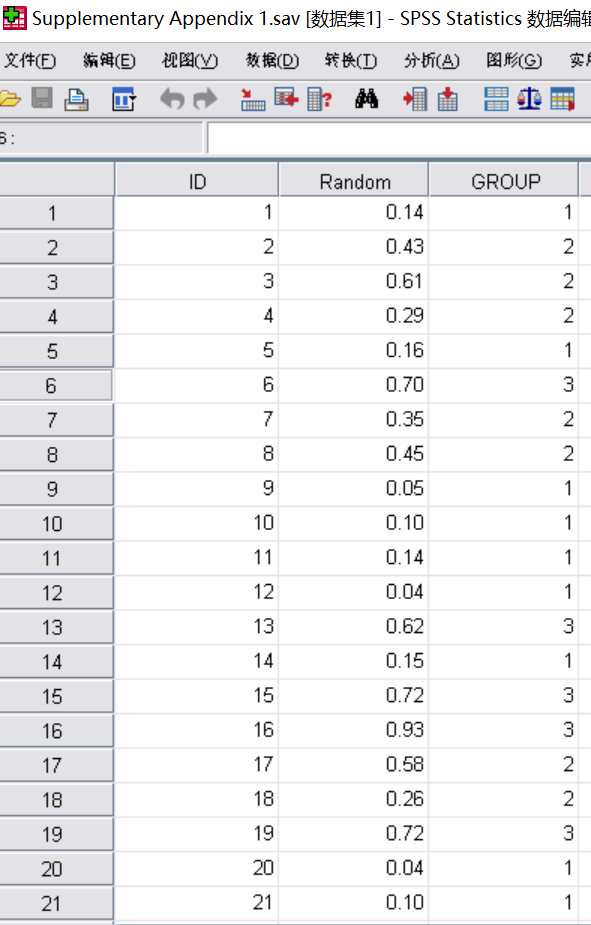


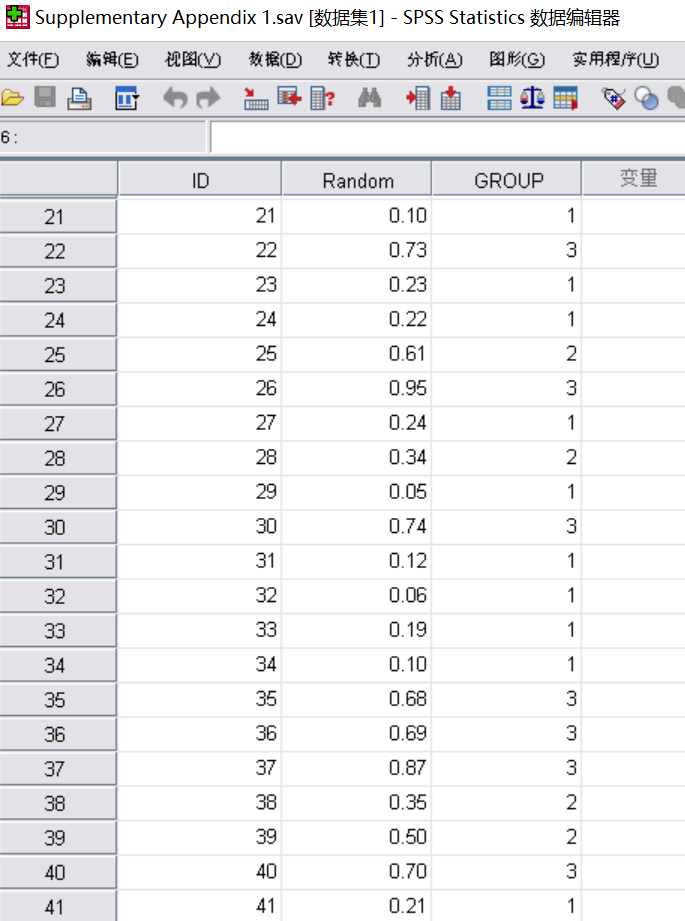


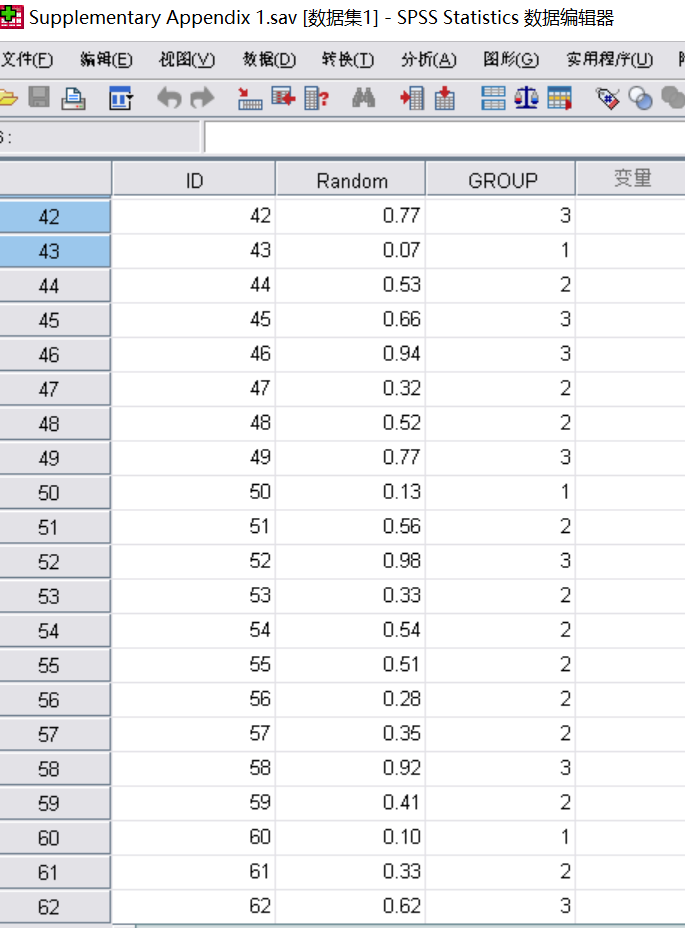


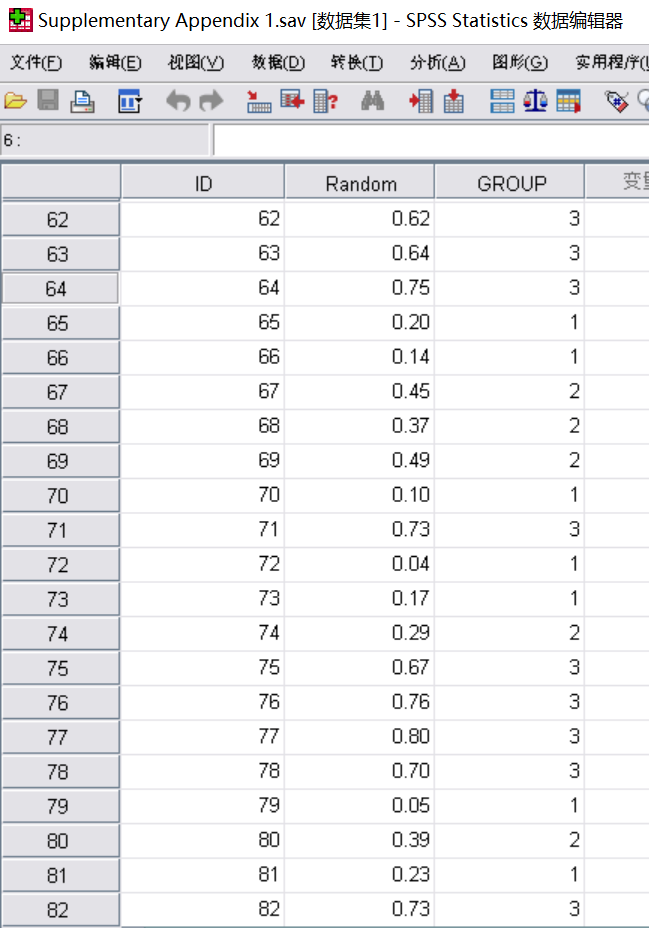


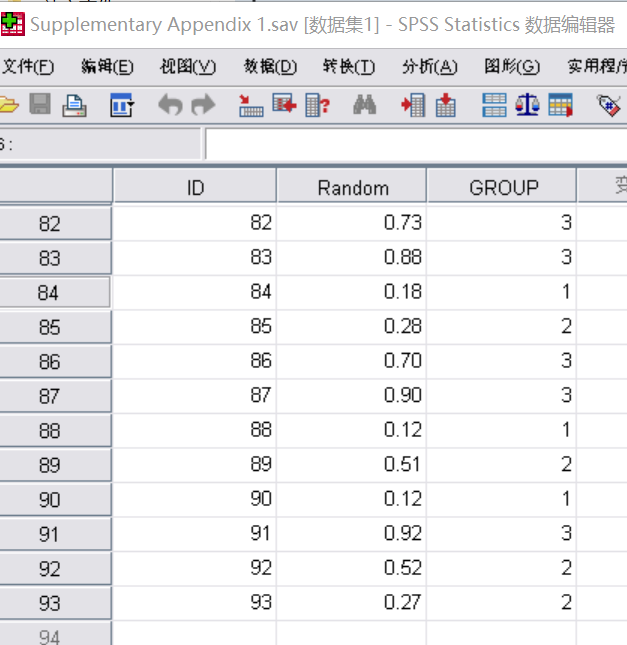

Supplement: Supplementary Materials — Supplementary 1. Supplementary Appendix 1: the results of randomization assignment. Supplementary 2. Supplementary Appendix 2: details of the training sessions. Supplementary 3. Supplementary Appendix 3: the general information questionnaire. Supplementary 4. Supplementary Appendix 4: the Connor–Davidson Resiliency Scale (C-D RS). Supplementary 5. Supplementary Appendix 5: the Nurses' Disaster Nursing Ability Assessment Scale. Supplementary 6. Table 1: general demographic data of the subjects. Supplementary 7. Table 2: scores of psychological ability and disaster nursing ability of the three groups of subjects before and after intervention (N = 93). Supplementary 8. Table 3: comparison of results before and after the training of emergency nurses in blank control group (N = 34). Supplementary 9. Table 4: comparison of results of emergency nurses before and after training in the intervention group (N = 31). Supplementary 10. Table 5: comparison of results before and after training of emergency nurses in the control group (N = 28). [file 6108057.f1.zip › Supplementary Appendix 1.docx]
